# A combination of strongly associated prothrombotic single nucleotide polymorphisms could efficiently predict venous thrombosis risk

**DOI:** 10.3389/fcvm.2023.1224462

**Published:** 2023-09-06

**Authors:** Shewaye Fituma Natae, Mohammed Abdulridha Merzah, János Sándor, Róza Ádány, Zsuzsanna Bereczky, Szilvia Fiatal

**Affiliations:** ^1^ Department of Public Health and Epidemiology, Faculty of Medicine, University of Debrecen, Debrecen, Hungary; ^2^ Doctoral School of Health Sciences, University of Debrecen, Debrecen, Hungary; ^3^ ELKH-DE Public Health Research Group, Department of Public Health and Epidemiology, Faculty of Medicine, University of Debrecen, Debrecen, Hungary; ^4^ Division of Clinical Laboratory Science, Department of Laboratory Medicine, Faculty of Medicine, University of Debrecen, Debrecen, Hungary

**Keywords:** venous thrombosis, risk prediction, single nucleotide polymorphisms (SNPs), cardiovascular risk (CVD), Hungarian population

## Abstract

**Background:**

Venous thrombosis (VT) is multifactorial trait that contributes to the global burden of cardiovascular diseases. Although abundant single nucleotide polymorphisms (SNPs) provoke the susceptibility of an individual to VT, research has found that the five most strongly associated SNPs, namely, rs6025 (*F5* Leiden), rs2066865 (*FGG*), rs2036914 (*F11*), rs8176719 (*ABO*), and rs1799963 (*F2*), play the greatest role. Association and risk prediction models are rarely established by using merely the five strongly associated SNPs. This study aims to explore the combined VT risk predictability of the five SNPs and well-known non-genetic VT risk factors such as aging and obesity in the Hungarian population.

**Methods:**

SNPs were genotyped in the VT group (*n* = 298) and control group (*n* = 400). Associations were established using standard genetic models. Genetic risk scores (GRS) [unweighted GRS (unGRS), weighted GRS (wGRS)] were also computed. Correspondingly, the areas under the receiver operating characteristic curves (AUCs) for genetic and non-genetic risk factors were estimated to explore their VT risk predictability in the study population.

**Results:**

rs6025 was the most prevalent VT risk allele in the Hungarian population. Its risk allele frequency was 3.52-fold higher in the VT group than that in the control group [adjusted odds ratio (AOR) = 3.52, 95% CI: 2.50–4.95]. Using all genetic models, we found that rs6025 and rs2036914 remained significantly associated with VT risk after multiple correction testing was performed. However, rs8176719 remained statistically significant only in the multiplicative (AOR = 1.33, 95% CI: 1.07–1.64**)** and genotypic models (AOR = 1.77, 95% CI: 1.14–2.73). In addition, rs2066865 lost its significant association with VT risk after multiple correction testing was performed. Conversely, the prothrombin mutation (rs1799963) did not show any significant association. The AUC of Leiden mutation (rs6025) showed better discriminative accuracy than that of other SNPs (AUC = 0.62, 95% CI: 0.57–0.66). The wGRS was a better predictor for VT than the unGRS (AUC = 0.67 vs. 0.65). Furthermore, combining genetic and non-genetic VT risk factors significantly increased the AUC to 0.89 with statistically significant differences (*Z* = 3.924, *p* < 0.0001).

**Conclusions:**

Our study revealed that the five strongly associated SNPs combined with non-genetic factors could efficiently predict individual VT risk susceptibility. The combined model was the best predictor of VT risk, so stratifying high-risk individuals based on their genetic profiling and well-known non-modifiable VT risk factors was important for the effective and efficient utilization of VT risk preventive and control measures. Furthermore, we urged further study that compares the VT risk predictability in the Hungarian population using the formerly discovered VT SNPs with the novel strongly associated VT SNPs.

## Introduction

Venous thrombosis (VT) is one of the three leading causes of cardiovascular disease (CVD)-related mortality with a significant genetic predisposition (1–3). It is a multifactorial trait that contributes to the global burden of CVD (4–7). In Europe, although overall CVD-related morbidity is decreasing, mortality remains substantially high. CVD is the leading cause of mortality in Europe, accounting for over 3.9 million deaths annually (8–10). Furthermore, approximately 60 million CVD premature deaths (death < 70 years) have been reported in Europe (10).

VT is a major health problem with a significant annual incidence (7.62/100,000) and mortality (3.70/100,000) (11). A higher burden of CVD-related mortality has been reported in Central and Eastern Europe (8, 12). Hungary shares the highest proportion of this mortality (8, 13). CVDs remain the most prominent cause of death in Hungary (13). As of 2014, approximately 35,000 women and 27,000 men have died from CVDs annually, accounting for 55% and 45% of all deaths for women and men, respectively (13). The age-standardized CVD death rate in Hungary is more than double the European Union (EU) average reported in 2014 (13). The availability of prophylaxis could significantly avert this burden by targeting high-risk individuals for treatment (11, 14). According to Rudolf Virchow's triad explanation (15), thrombosis is the result of three major factors, namely, blood flow stasis (16, 17), endothelial injury (18–20), and hypercoagulability (21). The inheritable prothrombotic factors influence VT risk via the coagulation process (21, 22), whereas the non-inheritable risk factors influence VT risk either via stasis or endothelial injury (23, 24).

Various studies have established the impact of heritable factors on VT risk (25–27). The incidence of repeated hospitalization due to VT is twofold higher in people with affected families than that in the general population (1–3). Although abundant single nucleotide polymorphisms (SNPs) provoke the susceptibility of an individual to VT (28–31), research has found that the five most strongly associated SNPs, namely, rs6025 (Leiden mutation) in the *F5* gene, rs1799963 (prothrombin G20210A) in the coagulation factor 2 gene (*F2*), rs8176719 (non-O blood type) in the *ABO* gene, rs2036914 in the coagulation factor eleven gene (*F11*), and rs2066865 in the fibrinogen gamma gene (*FGG*), play the greatest role in determining VT incidence and recurrence in genetically vulnerable individuals (29, 32, 33).

The Leiden mutation is one of the most dominant inheritable VT risk factors that increase the burden of VT in genetically vulnerable individuals (34–36). The Leiden mutation/*F5* prevalence is unevenly distributed across Europe with an average prevalence of 4% in the general population. The highest frequency is reported in Southeastern Europe and Northern Europe, whereas the lowest frequency is reported in Eastern and Western Europe (37, 38). The Leiden mutation prevalence is highest in European descent populations (3%–8%) (34, 39), followed by Caucasian Americans (5%). However, it is highly rare in African Americans (1.2%) and Asian-Americans (0.45%) (34) and absent in Africans (40, 41). Similarly, the prothrombin gene mutation/*F2*, often known as the G20210A mutation, is the second most prevalent inheritable VT risk in Caucasians (2%–4%) (39), particularly those of European ancestry (4%) and Caucasian Americans (2%). However, it is less prevalent in African Americans, accounting for approximately 0.4% (one in 250), and highly rare in Africans and Asians (39, 42).

Often, due to their coexistence and possible gene‒gene interaction, the prothrombin gene mutation (rs1799963) and Leiden mutation (rs6025) SNPs were studied together (43). Furthermore, studies showed that the ancestral distribution of coagulation factor 11 (rs2036914) is similar in both Caucasians and African Americans (44, 45). Studies indicated that O blood-type individuals are at lower risk of VT than non-O blood-type individuals (46, 47), who are at a higher risk of VT (48–51). In addition, Kinsella et al. reported that the risk of venous thromboembolism (VTE) is higher in African Americans and non-O blood-type individuals than that in Caucasians and O blood-type individuals (52).

An individual who is a carrier of multiple variants is more vulnerable to VT. Studies have indicated that the combination of strongly associated VT SNPs [rs6025 (*F5*), rs1799963 (*F2*), rs8176719 (*ABO*), rs2036914 (*F11*), and rs2066865 (*FGG*)] poses a greater VT risk than that risk occurring by an individual SNP (29, 53). The genetic risk score (GRS) of strongly associated VT variants results in the greatest risk compared with a larger number of SNP combinations. De Haan et al. (29) showed that the VT risk prediction of the 5-SNP risk score is equivalent to that of the 31-SNP risk score.

In addition, studies have indicated that dual exposure to VT risk factors (genetic and non-genetic) increases the susceptibility of an individual to VT (4, 29). Aging and obesity are well-known non-inheritable VT risk factors that hasten the onset of VT (29). As a result of multiple anatomical and pathophysiological changes, the elderly are prone to age-related cardiovascular morbidity and mortality (54–56). Aging plays a major role in the higher incidence of VT risk (1%) in elderly individuals (19, 54–57). The diminished efficiency of the calf muscle pump due to aging could lead to peripheral blood reflux and stasis resulting in thrombosis formation (54). Furthermore, age-related endothelial dysfunction is also a contributing factor to the higher incidence of VT in elderly individuals compared with that in younger individuals (19). Valve thickness, muscle fiber atrophy, and reduced endothelial anticoagulant properties are some pathophysiological changes that increase the VT risk among elderly individuals (54, 55). Correspondingly, obese individuals are at higher VT risk than normal-weight individuals. Previously conducted studies showed that the VT risk was two- to sixfold higher in obese individuals than that in normal-weight individuals [body mass index (BMI) = 20–24.9 kg/m^2^] (58–62). A study indicated that the VT risk was higher among aged (>50 years old) and obese individuals [61).

Stratifying higher-risk individuals based on their genetic profiling for thromboprophylaxis is important for efficient utilization of the available resources (29). Furthermore, the possibility of reducing unexpected consequences of massive supplementation of prophylactic treatment would be high (63). Although the 5-SNP impact on VT risk is huge, association and risk prediction models are rarely established by using merely five strongly associated SNPs. No study has yet been conducted to explore the VT risk predictability of the combined five strongly associated prothrombotic SNPs in the VT subjects from the Hungarian population. Consequently, this study aims to explore the VT risk predictability of the combined five SNPs [rs6025 (*F5* Leiden), rs2066865 (*FGG*), rs2036914 (*F11*), rs8176719 (*ABO*), and rs1799963 (*F2*)] in the Hungarian population.

## Methods and materials

### Study population

A total of 698 subjects were involved in the case‒control study, of which 298 were VT patients and 400 were healthy controls. The VT patients were recruited consecutively by the Division of Clinical Laboratory Science, Department of Laboratory Medicine, Faculty of Medicine, University of Debrecen during a 1-year period. VT diagnosis was established by standard diagnostic modalities, such as color Doppler ultrasound and phlebography at the Department of Internal Medicine. The controls were selected from the general population via a comprehensive health survey (see survey details and the created database elsewhere) and were free from VT according to a self-report questionnaire conducted 12 months prior to the survey (64).

### DNA isolation

DNA was extracted from the peripheral blood using a MagNA Pure LC system (Roche Diagnostics, Basel, Switzerland) with a MagNA Pure LC DNA Isolation Kit–Large Volume according to the manufacturer's instructions. The extracted DNA was eluted in a 200 μl MagNA Pure LC DNA Isolation Kit–Large Volume elution buffer.

### SNP selection and genotyping

Based on the genome-wide association study (GWAS) results (30, 65, 66) and our previously conducted studies (4, 67), we identified and considered the five strongly associated prothrombotic SNPs, namely, rs6025 (*F5*), rs2066865 (*FGG*), rs2036914 (*F11*), rs8176719 (*ABO*), and rs1799963 (*F2*), in our current study. We considered them due to their confirmed large effect size and potential predictability of inheritable VT risk (29, 67). The assay design and genotyping were performed by the Karolinska University Hospital, Stockholm, Sweden, Mutation Analysis Core Facility (MAF). A MassARRAY platform (Sequenom, CA, USA) with iPLEX Gold chemistry was used for genotyping. Quality control, validation, and concordance analysis were conducted by the MAF.

### Genetic risk score

The weighted GRS (wGRS) and unweighted GRS (unGRS) were computed to identify the combined effect of the included SNPs on VT risk. In the GRS, the individuals were assigned based on the total number of risk-increasing alleles. Consequently, “0,” “1,” and “2” codes were given for the absence, heterozygosity, and homozygosity of risk alleles, respectively. When the risk allele was found to be protective, the coding for the homozygous risk allele became “0” and “2” for the other homozygous allele (67). Accordingly, the unGRS was simply calculated by adding all risk alleles in a given locus with the assumption that all alleles had the same effect. To comprehend the stronger relationship of some SNPs with VT, we also calculated the wGRS by assigning weights to the risk allele of each SNP corresponding to the logarithm of the average risk estimates reported in the previously conducted genetic association study (29).

Moreover, to determine which SNP is more influential in its discriminatory accuracy of the area under the receiver operating characteristic curve (AUC), we added each SNP one by one into a model. Therefore, we started with the SNP with the highest odds ratio (OR), i.e., the Leiden mutation (rs6025) in the *F5* gene, and assessed whether adding more SNPs in a model could improve the AUC. We continued adding all other SNPs into a model until we verified that adding more SNPs into a model could not reveal any significant discriminatory accuracy.

### Non-genetic VT risk factors

We considered age (≥60 years), sex, and obesity (BMI ≥ 30 kg/m^2^) as non-genetic VT risk factors. We included each non-genetic risk factor and their combination with genetic VT risk factors into a model to verify the difference in the AUC and their VT risk predictability in the study population. A logistic regression model was used to generate a combined risk score of genetic and non-genetic VT risk factors.

### Statistical analysis

Statistical tests were computed using the PLINK (version 1.9) and IBM SPSS (version 26.0) statistical software. The Mann‒Whitney *U*-test was used to compare the age, BMI, and GRS distribution in the study population. The Shapiro–Wilk normality test was used to test the distribution of quantitative variables. The Hardy–Weinberg equilibrium (HWE) and risk allele frequency differences between the VT group and control group were estimated using the *X*^2^ test. The association between the five SNPs and VT risk was assessed by the OR with their respective 95% confidence interval (CI) under all genetic models, namely, the multiplicative, additive, dominant, recessive, and genotypic models. Likewise, a logistic regression analysis was also used to compute the OR with 95% of individual SNPs and genetic, non-genetic, and combined VT risk factors.

In addition, the area under the receiver operating characteristic (ROC) curve was determined to assess how well its score classifies the VT group and control group. In general, the AUC ranged from 0.5 (no discrimination between the VT group and control group) to 1.0 (perfect discrimination). We compared the AUCs of the genetic, non-genetic, and combined risk models. The SPSS IBM version 26.0 was used to calculate the ROC curves and AUCs. The Bonferroni multiple testing correction was employed to prevent multiple comparison problems (0.05/5, *p* < 0.01). Statistically significant variables were declared at a conventional *p*-value of 0.05.

### Ethical approval

The Hungarian Scientific Council on Health Research committee approved the protocol (61327-2017/EKU). All participants provided written consent before their participation.

## Results

### Characteristics of the study participants

In total, 698 subjects were enrolled in the case–control study, of which 298 were VT patients and 400 were healthy controls. All subjects with complete genotypic and covariate data were considered for the analyses. The proportion of male participants (51%) in the VT group was higher than that in the control group (44%). The age distribution of the VT group was shifted toward the elderly group, and their mean age was significantly higher than that of the control group (63.4 ± 16.4 vs. 43.8 ± 12.6 years, *p* < 0.001) (Supplementary Figure S1). However, the distribution of BMI values (kg/m^2^) did not differ significantly (28.2 ± 8.2 vs. 27.2 ± 5.5, *p* = 0.76). The marker check and detailed information of each SNP, including rs (SNP identifier), base pair position (BP), chromosome number (CHR), and major and minor alleles, are listed in Table 1.

**Table 1:** Marker check of the selected SNPs in the study.

Gene	*F5*	*FGG*	*F11*	*ABO*	*F2*
rs ID	rs6025	rs2066865	rs2036914	rs8176719	rs1799963
BP	169549811	154604124	186271327	133257521	46739505
CHR	1	4	4	9	11
Alleles (major: minor)	C:T	G:A	T:C	C:DEL	G:A
Genotype %	100%	100%	100%	100%	100%
MAF	0.1254	0.2536	0.5745	0.4441	0.02436
O(HET)	0.2077	0.3954	0.4957	0.4814	0.04871
E(HET)	0.2193	0.3786	0.4889	0.4938	0.04752
HWE *p*-value	*0.1665*	*0.2716*	*0.7569*	*0.5396*	*1*

MAF, minor allele frequency; O(HET), observed heterozygosity; E(HET), expected heterozygosity.

### Risk allele frequency comparison in the study population

The genotypic results were available for 698 subjects: VT patients (*n* = 298) and healthy controls (*n* = 400). All SNPs were tested to determine whether the observed allele frequencies were in accordance with the HWE; no significant deviation from the HWE was detected in the study population (Table 1). The risk allele frequencies of the five prothrombotic SNPs analyzed in the study are listed in Table 2. The risk allele frequencies of rs6025 (*F5*), rs2036914 (*F11*), and rs8176719 (*ABO*) were higher in the VT group than those in the control group, and the differences remained statistically significant after multiple testing correction was performed (*p < 0.01*) (Table 2).

**Table 2:** Risk allele frequency comparison of the VT group and control group in the Hungarian population.

Gene	SNP	A1	Cases	Controls	A2	*X* ^2^	OR (95% CI)	*p*-value
			(*n* = 298)	(*n* = 400)				
*F5*	rs6025	T	0.203	0.0675	C	57.21	3.52 (2.50–4.95)	<0.001*
*FGG*	rs2066865	A	0.2836	0.2312	G	4.94	1.32 (1.03–1.68)	0.026
*F11*	rs2036914	C	0.6191	0.5412	T	8.47	1.38 (1.11–1.71)	0.003*
*ABO*	rs8176719	C	0.5956	0.5262	DEL	6.66	1.33 (1.07–1.64)	0.001*
*F2*	rs1799963	A	0.0302	0.02	G	1.50	1.53 (0.77–3.02)	0.221

A1, risk allele; A2, reference allele; *X*^2^, chi-square.

* *p* < 0.01 considered significant after multiple correction testing.

In addition, we also computed the protective allele frequency of the *ABO* gene (DEL); its frequency was higher in the control group than that in the VT group (Supplementary Table S1).

### Association between SNPs and VT risk in the study population using genetic association models

The association strengths regarding VT risk using complete genetic association models (multiplicative, additive, dominant, recessive, and genotypic models) were estimated. Only the Leiden mutation (rs6025) and *F11* (rs2036914) remained significant after adjustment for multiple testing correction (*p* < 0.01). In particular, the Leiden mutation variant strongly influenced the VT risk in the Hungarian population (*p* < 0.001): among the patients with VT due to the Leiden mutation, the OR of VT risk ranged from 3.25 (heterozygous genotypic for risk variant; OR = 3.25, 95% CI: 2.22–4.76) to 19.67 (OR = 19.6, 95% CI: 2.57–150.4) in the recessive model/ those who were homozygous for risk variant.

The rs8176719 (*ABO*) remained statistically significant only in the multiplicative (OR = 1.33, 95% CI: 1.07–1.64**)** and genotypic models (OR = 1.77, 95% CI: 1.14–2.73); nevertheless, it lost its significance in other models after adjustment for multiple testing correction. Similarly, the rs8176719 (*ABO*) protective variant remained statistically significant only in the multiplicative model (OR = 0.75, 95% CI: 0.61–0.93) (Supplementary Table S2). In addition, the *FGG* (rs2066865) expressed a significant association with VT risk in the multiplicative, additive, and dominant models before multiple testing correction; however, it lost its significance after adjustment was performed. Conversely, the *F2* (rs1799963) did not show any statistically significant association with VT directly with any of the used models (Table 3).

**Table 3:** Genetic association test results in the VT group and control group of the study population: implication to determine the inheritable VT disease risk factors in the Hungarian population.

Model	Gene	*F5*	*FGG*	*F11*	*ABO*	*F2*
	SNP	rs6025	rs2066865	rs2036914	rs8176719	rs1799963
Multiplicative	*X* ^2^	57.21	4.94	8.47	6.66	1.50
	OR (95% CI)	3.52 (2.50–4.95)	1.32 (1.03–1.68)	1.38 (1.11–1.71)	1.33 (1.07–1.64)	1.53 (0.77–3.02)
	*p*	<0.001	0.026	0.004	0.001	0.221
Additive	*X* ^2^	54.35	5.17	8.59	6.50	1.53
	OR (95% CI)	3.52 (2.50–4.95)	1.32 (1.03–1.68)	1.38 (1.11–1.71)	1.33 (1.07–1.64)	1.53 (0.77–3.02)
	*p*	<0.001	0.02302	0.003	0.011	0.216
Dominant	*X* ^2^	49.61	4.32	5.71	4.88	1.53
	OR (95% CI)	3.67 (2.52–5.33)	1.38 (1.02–1.86)	1.64 (1.09–2.47)	1.54 (1.04–2.26)	1.543
	*p*	<0.001	0.038	0.017	0.03	0.215
Recessive	*X* ^2^	16.07	2.1	5.72	3.96	
	OR (95% CI)	19.67 (2.57–150.4)	1.61 (0.84–3.08)	1.47 (1.07–2.03)	1.39 (1.00–1.91)	
	*p*	<0.001	0.147	0.017	0.047	NA
Genotypic	*X* ^2^	54.35	5.18	8.64	6.60	1.534
	OR (95% CI)	3.25 (2.22–4.76)^a^	1.81 (0.94–3.51)	1.96 (1.24–3.08)	1.77 (1.14–2.73)	1.54 (0.77–3.07)
	*p*	<0.001	0.075	0.013	0.01	0.215

^a^ CT (heterozygous for a risk variant), NA = the value of one cell is 0, i.e., <5; hence, the *X*^2^ test is not applicable.

### Comparison of genetic risk scores in the study population

**Figure 1:**
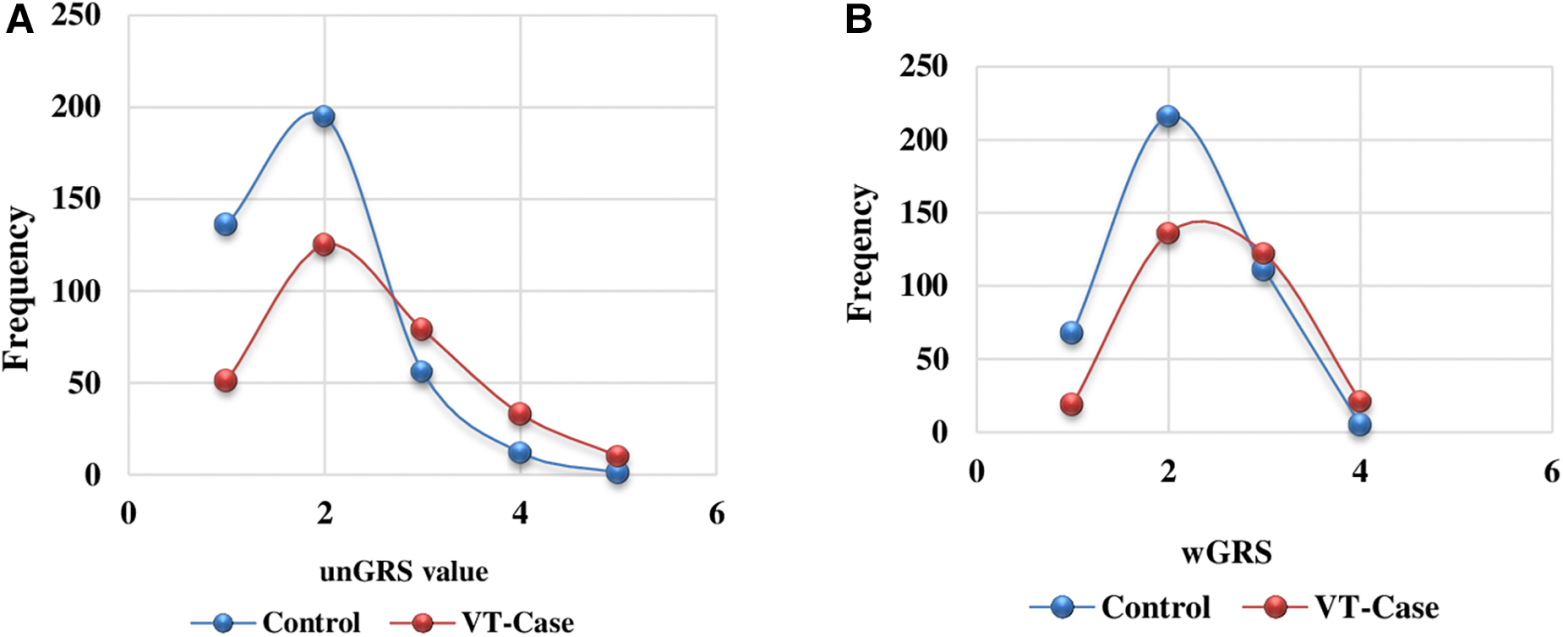
Unweighted (**A**) and weighted (**B**) GRS distribution comparison among the VT group and the control group of the Hungarian population.

The unGRS and wGRS of the five SNPs were computed for the 298 VT patients and 400 healthy controls. The unGRS ranged from 0 to 6 (3.46 ± 1.31) and 0 to 7 (2.77 ± 1.28) for the VT and control groups, respectively (Figure 1A). The wGRS ranged from 0 to 4.6 (1.93 ± 0.97) and 0 to 4.7 (1.37 ± 0.78) for the VT and control groups (Figure 1B).

### Association of GRS with VT risk

The distributions of other covariate variables including the wGRS and unGRS were significantly distinct (*p* < 0.001) between the two groups (Supplementary Table S3). The test revealed significant differences (case vs. control) in age (median = 65; 44, *p* < 0.001), BMI (median = 28.72; 26.75, *p* < 0.001), unGRS (median = 3; 3, *p* < 0.001), and wGRS (median = 1.79; 1.34, *p* < 0.001). Although the median unGRS values for the VT group and control group were similar, a larger unGRS value was more frequent in the VT group than in the control group.

Table 4 lists the multivariate logistic regression analysis results of covariate variables adjusted for sex and age. Of the well-known non-genetic VT risk factors, age and obesity were significantly associated with VT risk in the study population, which was higher in the VT group than that in the control group (Table 4). The VT risk was 12.8 times higher in the elderly subjects aged ≥60 years than that among the subjects aged below 60 years (AOR = 12.83, 95% CI: 8.38–19.63). Similarly, the VT risk was 2.3 times higher in the obese subjects **(**BMI > 30 kg/m^2^) than that in the normal-weight subjects (AOR = 2.28, 95% CI: 1.51–3.42). Furthermore, the wGRS remained statistically significant after we adjusted for both sex and age (AOR = 2.69, 95% CI: 1.74–4.19 and AOR = 2.24, 95% CI: 1.51–3.32, respectively). However, the unGRS lost its statistical significance (AOR = 0.88, 95% CI: 0.65–1.18) in the multivariate regression analysis model (Table 4).

**Table 4:** Association of covariate variables with VT risk in the Hungarian population.

Variables	VT risk^b^			VT risk^c^	
	ß	*p*-value	COR (95% CI)	AOR (95% CI)^d^	AOR (95% CI)^e^
Sex (male)^a^	0.271	0.077	1.31 (0.97–1.77)	—	1.16 (0.84–1.61)
Age ≥ 60 years	2.468	<0.001	11.79 (7.96–17.49)	12.83 (8.38–19.63)*	—
BMI (≥30 kg/m^2^)	0.850	<0.001	2.34 (1.59–3.45)	1.41 (0.88–2.26)	2.28 (1.51–3.42)*
unGRS	0.412	<0.001	1.51 (1.34–1.71)	0.88 (0.65–1.18)	0.94 (0.72–1.21)
wGRS	0.729	<0.001	2.07 (1.72–2.50)	2.69 (1.74–4.19)*	2.24 (1.51–3.32)*

^a^ Female is a reference.

^b^ Crude odds ratio (COR).

^c^ Adjusted odds ratio (AOR).

^d^ Adjusted for sex.

^e^ Adjusted for age.

* *p*-value < 0.0001.

### VT risk prediction in the study population

**Figure 2:**
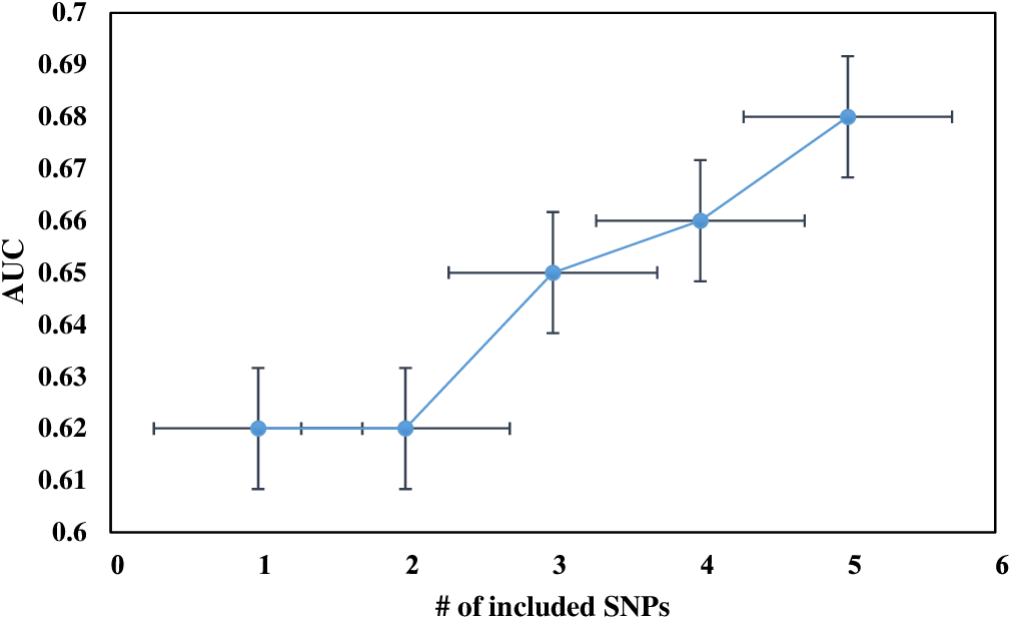
The change in the discriminatory accuracy of the AUC of the genetic risk score after adding each SNP into a model; we started with the Leiden mutation (rs6025) with the highest effect size and ended with rs2036914 (*F11*) with the lowest effect size of the five SNPs. The addition of each SNP increases the AUC value after *F2* (rs1799963).

We calculated the ROC curve to assess how well the score classified VT in the case and control groups. The AUC of the SNPs ranged from 0.51 (95% CI: 0.47–0.55, *p* = 0.64) for rs1799963 in *F2* to 0.62 (95% CI: 0.57; 0.66, *p* < 0.001) for rs6025 in *F5*. The discriminative accuracy of the model improved by adding each SNP (Figure 2). We started with the Leiden mutation (rs6025) with the highest effect size and ended with rs2036914 (*F11*) with the lowest effect size among the five SNPs. The addition of each SNP increased the AUC after *F2* (rs1799963).

The AUC of the 5-SNP risk score was 0.68 (95% CI: 0.64; 0.72). The variability proportion explained by the Leiden mutation (rs6025) was higher than that of the 5-SNP risk score (8% vs. 7%). Furthermore, approximately 39% of the variability observed was attributed to the combination of genetic and non-genetic risk factors, which is higher than that of those factors independently (Table 5). Similarly, the ROC curve for the weighted 5-SNP risk score had an AUC of 0.68 (95% CI: 0.64–0.72), i.e., there was a 68% probability that a randomly selected VT patient will have a higher score than that of a randomly selected control subject. The wGRS was a better predictor for VT than the unGRS (AUC = 0.65, 95% CI: 0.60–0.69).

**Table 5:** Venous thrombosis risk predictability of the Leiden mutation, genetic risk, non-genetic risk, and combined model in the Hungarian population.

Variables	*r* ^2^	*N* = 698	
		AUC (95% CI)	*p*-value
Leiden mutation risk model^a^	0.08	0.62 (0.57–0.66)	<0.0001
Genetic risk model^b^	0.09	0.68 (0.64–0.72)	<0.0001
Non-genetic risk model^c^	0.31	0.85 (0.82–0.88)	<0.0001
Combined model^d^	0.39	0.89 (0.86–0.91)	<0.0001
Difference^e^	—	0.039 (0.02–0.059)	<0.0001

*r*^2^: variability explained by each variable.

A total of 298 VT patients and 400 healthy controls with complete genotypic and covariate data were considered during the analysis.

^a^ Leiden mutation, the most prevalent inheritable VT risk variant in the study population.

^b^ **Genetic risk model**: weighted GRS computed from the five SNPs (rs6025, rs2066865, rs2036914, rs8176719, and rs1799963).

^c^ **Non-genetic risk model:** age [5-year interval, sex, and BMI (<25, 25–30, and ≥30 kg/m^2^)].

^d^ **Combined risk model:** genetic risk model plus non-genetic risk model.

^e^ Difference between the combined and non-genetic risk models.

There was a difference between the discriminative accuracy of the 5-SNP risk score in men (AUC = 0.68, 95% CI: 0.62–0.74, *p* < 0.001) and women (AUC = 0.61: 95% CI: 0.55–0.67, *p* < 0.001). Moreover, the AUC of the wGRS of the 5-SNPs was significantly higher in men (AUC = 0.71, 95% CI: 0.65–0.76, *p* < 0.001) than that in women (AUC = 0.63, 95% CI: 0.57–0.69, *p* < 0.001).

### Risk prediction based on a combination of genetic and non-genetic risk factors

**Figure 3:**
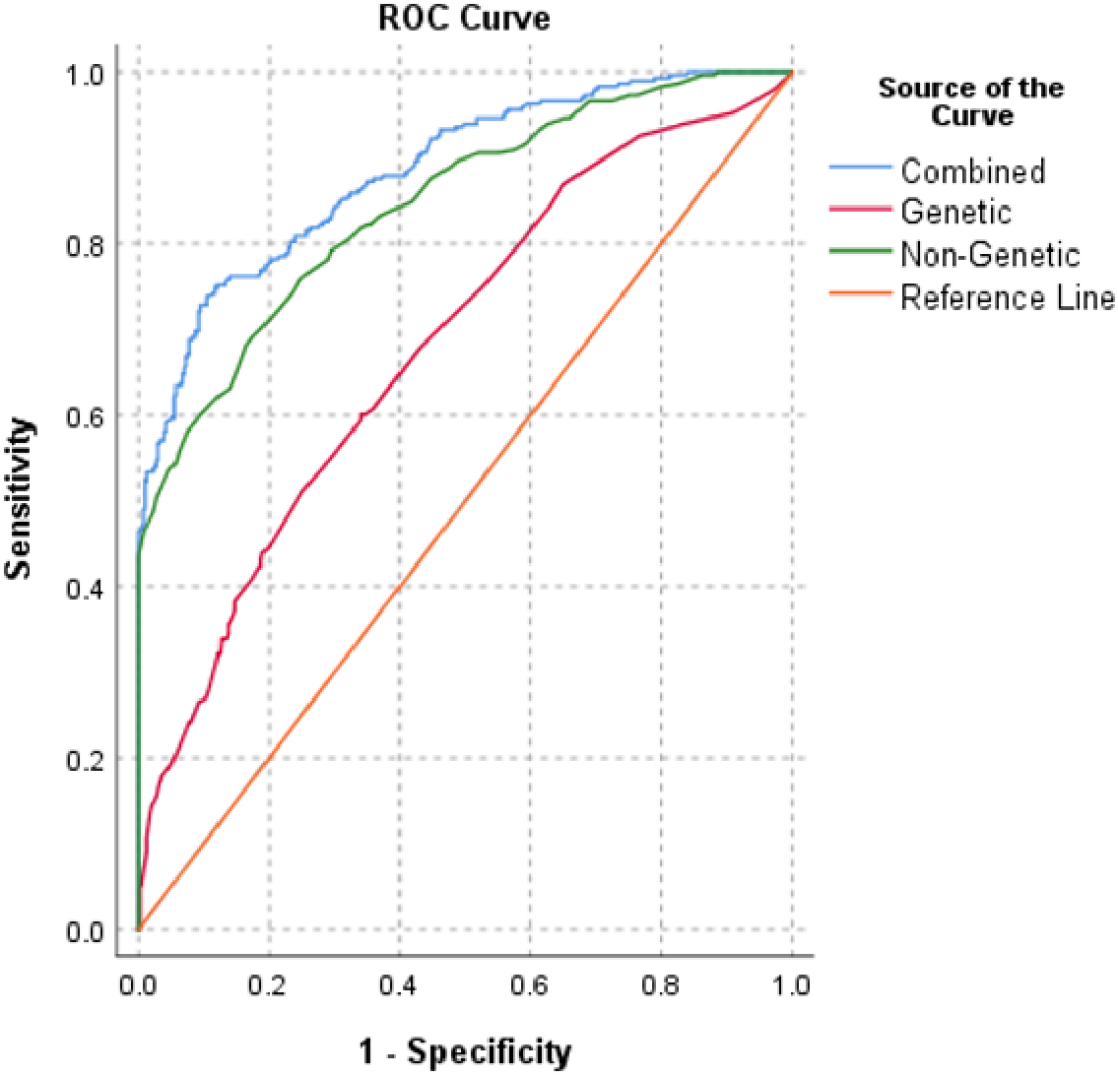
ROC curves for combined (genetic and non-genetic), genetic (five prothrombotic SNPs), and non-genetic (age, sex, BMI) risk models.

We also evaluated the discriminative accuracy of well-known non-genetic VT risk factors such as age, sex, and obesity to explore their independent and combined VT risk predictability in the study population. The independent AUCs of age and obesity were 0.84, *p* < 0.0001, and 0.59, *p* < 0.001, respectively. The combination of all well-known VT risk factors changed the discriminative accuracy of the AUC to 0.85, *p* < 0.000. Similarly, when we added the non-genetic risk factors into the genetic risk factors, the AUC significantly projected to 0.89 (95% CI: 0.86–0.91) compared to that in the genetic (AUC = 0.68) or non-genetic risk factor predictability (AUC = 0.85; *p* < 0.0001) (Figure 3). The AUC difference in the combined and non-genetic risk factors was statistically significant (AUC = 0.039, 95% CI: 0.02–0.059, *p* < 0.0001). There was no significant AUC difference between men and women in the non-genetic (men: AUC = 0.81, 95% CI: 0.76–0.86 vs. women: AUC = 0.82, 95% CI: 0.78–0.87) and combined risk score (men: AUC = 0.87, 95% CI: 0.83–0.91 vs. women: AUC = 0.86, 95% CI: 0.82–0.90) models.

## Discussion

Although there are abundant SNPs that provoke VT events in genetically vulnerable individuals, the contributions of the five strongly associated SNPs, namely, rs6025 (*F5*), rs2066865 (*FGG*), rs2036914 (*F11*), rs8176719 (*ABO*), and rs1799963 (*F2*), to VT risk is immensely high (29, 32, 33). Moreover, previously conducted studies demonstrated the importance of these five prothrombotic SNPs in the relapse of inheritable VT (29, 32, 33). Consequently, we aimed to explore the combined genetic risk predictability of strongly associated VT SNPs and well-known non-genetic VT risk factors in the Hungarian population. Thus, stratifying high-risk individuals based on their genetic profiling might help for the efficient utilization of scarcely available thromboprophylaxis, which might reduce the premature death attributed to VT and CVDs.

In our present study, we considered five VT-associated SNPs to explore the genetic background of VT risk in the Hungarian population. Only the three SNPs, namely, rs6025 (*F5*), rs2036914 (*F11*), and rs8176719 (*ABO*), remained statistically significant after adjustment for multiple testing correction (*p* < 0.01). The highest VT risk was detected among the Leiden mutation carriers/rs6025 (OR = 3.52, 95% CI: 2.50–4.95). Its allele frequency was approximately threefold higher in the VT group (20%) than that in the control group (6.8%). Our findings were consistent with the finding of the previously conducted studies showing that the odds of VT risk are 3.5 (28) and 4.38 times higher for rs6025 (*F5*) variant carriers than those of non-carriers (68).

Moreover, numerous studies suggest that the Leiden mutation is vastly prevalent in Caucasians, particularly those of European descent. It is one of the most influential inheritable VT risk factors that increase the burden of VT in genetically vulnerable individuals (34–36). These findings support our study’s result that *F5* is highly prevalent in the case group (20%). Furthermore, it was strongly associated with the trait in all genetic association models. This highlights the fact that the Leiden mutation is an independent predictor of VT risk (28, 35, 69) and its contribution to the burden of VT is remarkable (69), particularly for genetically susceptible individuals and Caucasians (34). Likewise, the risk allele frequency and VT risk for rs2036914 (*F11*) and rs8176719 (*ABO*) were higher in the case group even after adjustment for multiple testing correction. A previously conducted study revealed that *F11* (rs2036914) is an independent predictor of VT (70), which is supported by our result that the risk was 1.38 times higher in the case group than that in the control group. Studies showed that VT risk distribution due to *F11* (rs2036914) is similar in Caucasians and African Americans (44, 45).

The allele frequency of the *ABO* SNP (rs8176719) was more prevalent in the control group (47.4% vs. 40.4%). In addition, it was revealed that the VT risk was lower (OR =  0.75, 95% CI: 0.61–0.93) among the subjects with the rs8176719 variant. Furthermore, the VT risk was 1.33 times higher in the risk variant carriers. Studies indicated that the O blood-type individuals are at a lower VT risk compared with the non-O blood-type individuals (46, 47). On the other hand, several studies showed that non-O blood-type individuals (A, AB, and B) were at a higher VT risk compared with non-O blood-type individuals (48–51). Our findings were also consistent with those of the previously conducted studies. Fang et al. reported that the VTE risk is higher in African Americans and non-O blood-type individuals than that in Caucasians and O blood-type individuals (52).

Although the risk allele frequencies and VT risk were not distinct in the case of prothrombin mutation (rs1799963), it was the second most prevalent risk variant in the Hungarian population. The reason for the lack of statistical significance despite the large OR might be attributed to the limited number of our VT patients. Our findings were also in line with those of the previously conducted studies showing that rs1799963 is more prevalent in European descent populations than in others (Americans, African Americans, Asians, and Africans) (23). Several studies showed that approximately two- to fourfold VT risk was attributed to a hypercoagulability state that resulted from a mutation in the prothrombin gene/rs1799963 (35, 71, 72).

Studies indicated that pooled variants have more impact on VT risk determination than a single variant (29, 53); consequently, we computed the wGRS and unGRS to determine the VT risk in the study population. Our findings showed that the wGRS is an independent predictor of VT risk in the study population, and its value was 2.37 times higher in the VT group than in the control group. Previously conducted studies also supported our findings (29, 53).

The impact of non-genetic risk factors on VT risk is also appreciable. Our study showed that VT was more prevalent in elderly (≥ 60 years) subjects (58.1% vs. 10.5%; *p < 0.0001*). Likewise, the odds of VT risk for elderly subjects were 12-fold higher than that of those aged <60 years. The VT risk increases with age due to different factors, such as anatomical (54), pathophysiological (54–57), and hormonal derangement (73). Consequently, it hastens and increases the vulnerability of elderly subjects to VT risk and other CVDs (19, 55–57). Furthermore, our findings showed that the VT risk is 2.28 times higher in the obese subjects than that in the normal-weight subjects. This finding is in line with those of the previously conducted studies that showed that obesity is an independent predictor of VT risk (58–62).

The ability to predict the risk of a certain event before its occurrence is important in clinical epidemiology. Precise risk prediction helps control an event at as early a stage as possible (74–77) and offers to use the available resources effectively and efficiently (74–77). We used the ROC curves to establish individual and combined VT risk predictability of the SNPs and non-inheritable VT risk factors to develop a risk stratification tool.

In our study, the highest AUC was obtained for the Leiden mutation (AUC = 0.62), whereas the lowest AUC was obtained for the prothrombin mutation/*F2* (0.52). The addition of each SNP into the model after *F5* increased the AUC in general. Our finding is in line with that of the studies showing that adding more SNPs into the model increases the AUC to a certain extent, but after a certain level, the AUC does not change, despite adding more explanatory variables into the model (29, 78).

We also found that the wGRS is a better predictor of VT risk than individual SNPs (0.68 vs. 0.62) and their combination with non-genetic risk factors yields a larger AUC with higher discriminatory accuracy (AUC = 0.89). This finding is consistent with that of the previous studies showing that the combination of clinical and genetic risk factors increases the VT risk eight times more than either the genetic or the clinical model alone (79, 80).

### Strengths and limitations of our study

Our study tried to verify the VT risk predictability in the Hungarian population only by using strongly associated VT SNPs [rs6025 (*F5*), rs1799963 (*F2*), rs8176719 (*ABO*), rs2036914 (*F11*), and rs2066865 (*FGG*)] mainly relating to the recurrence and higher incidence of VT risk and well-known non-genetic VT risk factors (age, obesity, and sex), which helped distinguish the higher-risk individuals for the prevention and control of VT in the study population. Furthermore, our study indicated the possibility of efficiently and effectively utilizing the available resource for risk prediction in the given population. However, our study lacked the comparison of formerly identified strongly associated VT SNPs with the novel loci, which are strongly associated with VT risk as well (66, 81–83). As a result, we urged further study that considers the novel and strongly associated VT SNPs and the formerly identified SNPs and their comparison on the VT risk predictability in the Hungarian population.

Altogether, the Leiden mutation, *F11*, and *ABO* risk alleles are highly prevalent and strongly determine the VT risk in the Hungarian population. The pooled genetic risk variants are more influential than a single variant alone. The combined model is the best predictor of VT risk, so stratifying high-risk individuals based on their genetic profiling and well-known non-modifiable VT risk factors is important for the effective and efficient utilization of VT risk preventive and control measures. Furthermore, our study lacks the comparison of formerly identified VT SNPs with the novel SNPs, which are strongly associated with VT risk. This might provide new insight into the VT risk and its determinants in the Hungarian population. As a result, we urged further study that considers the novel and strongly associated VT SNPs and the formerly identified SNPs and their comparison on the VT risk predictability in the Hungarian population.

## Data Availability

The original contributions presented in the study are included in the article/Supplementary Material, further inquiries can be directed to the corresponding author.
