# Supplementary material for: A combination of strongly associated prothrombotic single nucleotide polymorphisms could efficiently predict venous thrombosis risk
Source: Front Cardiovasc Med. 2023 Sep 6;10:1224462. doi: 10.3389/fcvm.2023.1224462 (PMC10511882; doi:10.3389/fcvm.2023.1224462)
Supplement: Supplementary file 1 [file Datasheet1.docx]

**A combination of strongly associated prothrombotic single nucleotide polymorphisms could efficiently predict venous thrombosis risk**

Shewaye Fituma Natae ^1,2^, Mohammed Abdulridha Merzah^1,2,^ János Sándor^1,3^, Róza Ádány^1^, Zsuzsanna Bereczky^4^, Szilvia Fiatal^1^

*^1^Department of Public Health and Epidemiology, Faculty of Medicine, University of Debrecen, 4032 Debrecen, Hungary*

*^2^Doctoral School of Health Sciences, University of Debrecen, 4032, Debrecen, Hungary*

*^3^ELKH-DE Public Health Research Group, Department of Public Health and Epidemiology, Faculty of Medicine, University of Debrecen, 4032 Debrecen, Hungary*

*^4^Division of Clinical Laboratory Science, Department of Laboratory Medicine, Faculty of Medicine, University of Debrecen, 4032 Debrecen, Hungary*

**Supplementary Materials**

1. **Supplementary Figure**

**Supplementary Figure 1:** Age distribution of VT patients and the control group of the Hungarian population.

1. **Supplementary Tables**

**Supplementary Table 1**: Risk allele frequency comparison among VT patients and healthy controls in the Hungarian population (the allele frequency of the O blood group variants was employed to determine the VT risk in the VT cases and controls)

| **Gene** | **SNP** | **A1** | **Cases**  **(N=298)** | **Controls**  **(N-400)** | **A2** | **CHISQ** | **OR(95% CI)** | ***p value*** |
| --- | --- | --- | --- | --- | --- | --- | --- | --- |
| F5 | rs6025 | T | 0.203 | 0.0675 | C | 57.21 | 3.52(2.50;4.95) | *<0.001** |
| FGG | rs2066865 | A | 0.2836 | 0.2312 | G | 4.937 | 1.32(1.03;1.68) | *0.026* |
| F11 | rs2036914 | C | 0.6191 | 0.5412 | T | 8.474 | 1.38(1.11;1.71) | *0.003** |
| ABO | rs8176719 | DEL | 0.4044 | 0.4738 | C | 6.661 | 0.75(0.61;0.93) | *0.001** |
| F2 | rs1799963 | A | 0.0302 | 0.02 | G | 1.496 | 1.53(0.77,3.020) | *0.221* |

***A1=risk allele, A2=reference allele, values in the cell indicate the frequencies of risk alleles in the case and control groups. *p<0.01 considered significant after multiple correction testing.***

**Supplementary Table 2:** Genetic association test results in the VT cases and control groups of the study population: implication to determine the inheritable venous thrombosis disease risk factors in the Hungarian population. [O blood group/protective blood group variants of the ABO gene were considered in this model to determine the VT risk among O blood group and non-O blood group subjects)

| **Model** | **Gene** | **F5** | **FGG** | **F11** | **ABO** | **F2** |
| --- | --- | --- | --- | --- | --- | --- |
|  | ***SNP*** | ***rs6025*** | ***rs2066865*** | ***rs2036914*** | ***rs8176719*** | ***rs1799963*** |
| **Multiplicative**  **Model** | X^2^ | 57.21 | 4.94 | 8.47 | 6.66 | 1.50 |
|  | OR(95% CI) | 3.52(2.50;4.95) | 1.32(1.03;1.68) | 1.38(1.11;1.71) | 0.75(0.61;0.93) | 1.53(0.77;3.02) |
|  | *p* | ***<0.001*** | *0.026* | ***0.004*** | ***0.001*** | *0.221* |
| **Additive**  **Model** | X^2^ | 54.35 | 5.17 | 8.59 | 6.50 | 1.53 |
|  | OR(95% CI) | 3.52(2.50;4.95) | 1.32(1.03;1.68) | 1.38(1.11;1.71) | 0.75(0.61;0.93) | 1.53(0.77;3.02) |
|  | *p* | ***<0.001*** | *0.02302* | ***0.003*** | ***0.011*** | *0.216* |
| **Dominant**  **Model** | X^2^ | 49.61 | 4.32 | 5.71 | 3.96 | 1.53 |
|  | OR(95% CI) | 3.67(2.52;5.33) | 1.38(1.02;1.86) | 1.64(1.09;2.47) | 0.72(0.52;0.99) | 1.54 |
|  | *p* | ***<0.001*** | *0.038* | ***0.017*** | *0.047* | *0.215* |
| **Recessive**  **Model** | X^2^ | 16.07 | 2.1 | 5.72 | 4.88 |  |
|  | OR(95% CI) | 19.67(2.57;150.4) | 1.61(0.84;3.08) | 1.47(1.07;2.03) | 1.54(1.05;2.26) |  |
|  | *p* | ***<0.001*** | *0.147* | ***0.017*** | *0.027* | *NA* |
| **Genotypic**  **Model** | X^2^ | 54.35 | 5.18 | 8.64 | 6.60 | 1.53 |
|  | OR(95% CI) | 3.25(2.22;4.76)* | 1.81(0.94;3.51) | 1.96(1.24;3.08) | 0.57(0.37;0.88) | 1.54(0.77;3.07) |
|  | *p* | ***<0.001*** | *0.075* | ***0.013*** | *0.037* | *0.215* |

****CT (heterozygous for a risk variant), NA= the value of one cell is 0 i.e <5 hence, the X^2^ test is not applicable.***

**Supplementary Table 3**: Mann‒Whitney U test of nonnormally distributed covariate variables of the study population.

| **Variables** | **Median** | | ***p value*** |
| --- | --- | --- | --- |
|  | **Cases(n=298)** | **Controls(n=400)** |  |
| Age | 65 | 44 | *<0.001* |
| BMI | 28.72 | 26.75 | *<0.001* |
| unGRS^¶^ | 3 | 3 | *<0.001* |
| wGRS | 1.79 | 1.34 | *<0.001* |

***^¶^Skewed data with high proportion of the same median value (3) for both groups; however, the distribution is still higher for cases (35.5%) than control group (29.3%).***
